# The Microbiome of the *Maculinea*-*Myrmica* Host-Parasite Interaction

**DOI:** 10.1038/s41598-019-44514-7

**Published:** 2019-05-29

**Authors:** Marco Di Salvo, Matteo Calcagnile, Adelfia Talà, Salvatore Maurizio Tredici, Massimo E. Maffei, Karsten Schönrogge, Francesca Barbero, Pietro Alifano

**Affiliations:** 1 Department of Biological and Environmental Sciences and Technologies, University of Salento, Lecce, 73100 Italy; 2 Department of Life Sciences and Systems Biology, University of Turin, Turin, 10123 Italy; 3 Centre for Ecology and Hydrology, Wallingford, OX10 8BB United Kingdom

**Keywords:** Coevolution, Microbial communities

## Abstract

*Maculinea* (=*Phengaris*) are endangered butterflies that are characterized by a very complex biological cycle. *Maculinea* larvae behave as obligate parasites whose survival is strictly dependent on both particular food plants and species-specific *Myrmica* ants. In this interaction, *Maculinea* caterpillars induce *Myrmica* workers to retrieve and rear them in the nest by chemical and acoustic deception. Social insect symbiotic microorganisms play a key role in intraspecific and interspecific communication; therefore, it is possible that the *Maculinea* caterpillar microbiome might be involved in the chemical cross-talk by producing deceptive semiochemicals for host ants. To address this point, the microbiota of *Maculinea alcon* at different larval stages (phytophagous early larvae, intermediate larvae, carnivorous late larvae) was analyzed by using 16S rRNA-guided metabarcoding approach and compared to that of the host ant *Myrmica scabrinodis*. Structural and deduced functional profiles of the microbial communities were recorded, which were used to identify specific groups of microorganisms that may be involved in the chemical cross-talk. One of the most notable features was the presence in all larval stages and in the ants of two bacteria, *Serratia marcescens* and *S*. *entomophila*, which are involved in the chemical cross-talk between the microbes and their hosts.

## Introduction

The endangered *Maculinea* butterflies (Lepidoptera, Lycaenidae) are perceived as “umbrella species” in many grassland ecosystems and have been chosen to raise support to conserve biodiversity in grassland habitats in Europe^1^. *Maculinea* butterflies are characterized by a complex life-history^2^. They are obligate parasites, and the survival of local populations is largely dependent on both specific food plants for the early instars and *Myrmica* ant species providing food and protection for the final instars^3^. Females of *Maculinea* lay eggs on a specific food plant, early larvae hatch and live feeding inside flowers for two to three weeks. As they reach their final instar, they drop to the ground and wait until they are collected by foraging workers of the genus *Myrmica* that return them to their nests. Inside the nests, the caterpillars of the five European species act either as predators, *Ma*. *arion* and *Ma*. *teleius*, preying directly on ant brood^4^ or adopt a “cuckoo” strategy, *Ma*. *alcon* and *Ma*. *rebeli* (which are considered as separate taxa according to Bonelli *et al*.^5^), as they mimic ant larvae and are fed directly by worker ants^3^. *Ma*. *nausithous* adopts both strategies^6^. Inside the ant nests, *Maculinea* last instars spend 11 (or 23 two-year developer^7^) months increasing their body mass before pupating inside the ant colony from which adults emerge the following summer.

Two steps are critical for the larval survival in *Maculinea* species: (i) the choice of suitable food plants for oviposition; and (ii) the interaction with the worker ants that adopt the larvae. Indeed, there is evidence that while *Maculinea* caterpillars can be rapidly adopted by any *Myrmica* species they encounter on the ground underneath the host plant, they tend to only survive in the nests of a single or a few species of *Myrmica*^8^. Several *Maculinea* species including *Ma*. *alcon*, the focus species of our study, may use different ant hosts in different European geographical regions^9^.

As *Maculinea* larvae leave their host plant, their profile of surface hydrocarbons is sufficiently similar to that of *Myrmica* spp. workers to ensure their adoption only by ants belonging to this genus^10^. Once they have been moved inside the host colony, the full integration of the parasites, particularly in “cuckoo” feeding species, is facilitated by both queen-like acoustic signals^11^ and more complex chemical mimicry matching the chemical profile of the primary *Myrmica* host ant species^12^. In colonies of non-host *Myrmica* species the *Maculinea* larvae are recognized as intruders and often killed^12^.

Besides the chemical and acoustic signaling, there is increasing evidence from social or non-social insects that symbiotic microorganisms may contribute to their nutrition, immune system modulation, and protection by the production of antibiotics or metabolites crucial for food digestion, detoxification, and nitrogen fixation^13^. Termites, ants, and bees harbor specialized communities of gut bacteria with beneficial functions^14–16^. Social interactions among individuals, such as the exchange of food and allogrooming, provide the opportunity to transfer and share these specialized gut communities^13^. In ants, horizontal transmission of microbes by trophallaxis might also occur between distinct species involved in tight interactions, as described for *Solenopsis invicta* and *Solenopsis richteri* parasitized by their inquiline ants, *Solenopsis daguerrei*^17^.

Gut microorganisms, through biosynthetic or catabolic pathways, may produce semiochemicals that function in the host as pheromones or kairomones. For instance, in the desert locust *Schistocerca gregaria*, *Pantoea agglomerans* and other common gut bacteria such as *Klebsiella pneumoniae* and *Enterobacter cloacae* metabolize plant-derived vanillic acid producing guaiacol and other components of the locust cohesion pheromone that is responsible for forming dense migrating swarms^18^. In contrast, in aphid guts *Staphylococcus sciuri* is the source of kairomones that are released into honeydew and act as attractants to aphid predators^19^. In the model organism *Drosophila melanogaster*, the diet-associated gut microbiome oversees important developmental, physiological and behavioral processes including mating attractiveness. Flies mate preferentially with individuals harboring the same microbiome assemblage, and mating preferences have been manipulated by introduction of selected gut microorganisms such as *Lactobacillus plantarum*, which is predominant in flies feeding on starch-rich substrates, into axenic flies^20^. Here mating preference was associated with the ability of the gut bacteria to affect the scent profile used by *Drosophila* in choosing mates^21^.

The interactions between microbiome assemblage and insect feeding strategy and behaviors have potential evolutionary implications, and may be considered as an important driver for insect speciation^20^. Recent studies have clearly shown that these interactions can be much more complex than imagined when they involve multiple trophic levels^22–25^. These studies prompted us to investigate whether the *M*. *alcon* caterpillar microbiome may be involved in the production of semiochemicals used to communicate with the *Myrmica* ants. Although the biology of *Maculinea* butterflies has been extensively studied, information on the associated microbial community is entirely lacking. We predict that in *Maculinea* “cuckoo” species, where chemical mimicry is crucial to obtain a full integration in the host colony, the stages associated with the ants possess characteristic microbial communities that are different from those found while they are feeding on the host plant. Due to the occurrence of food exchange (trophallaxis), we also expect some similarity in the microbiota of ants and fully-grown *Maculinea* larvae. To address these points, the microbiota of caterpillars at different larval stages were analyzed using 16S rRNA amplicon metabarcoding followed by microbiomes functional prediction, and compared to that of the host ant *My*. *scabrinodis*.

## Results and Discussion

### 16S rRNA-based profiling of bacterial communities from *Myrmica scabrinodis* and *Maculinea alcon* larvae

**Figure 1:**
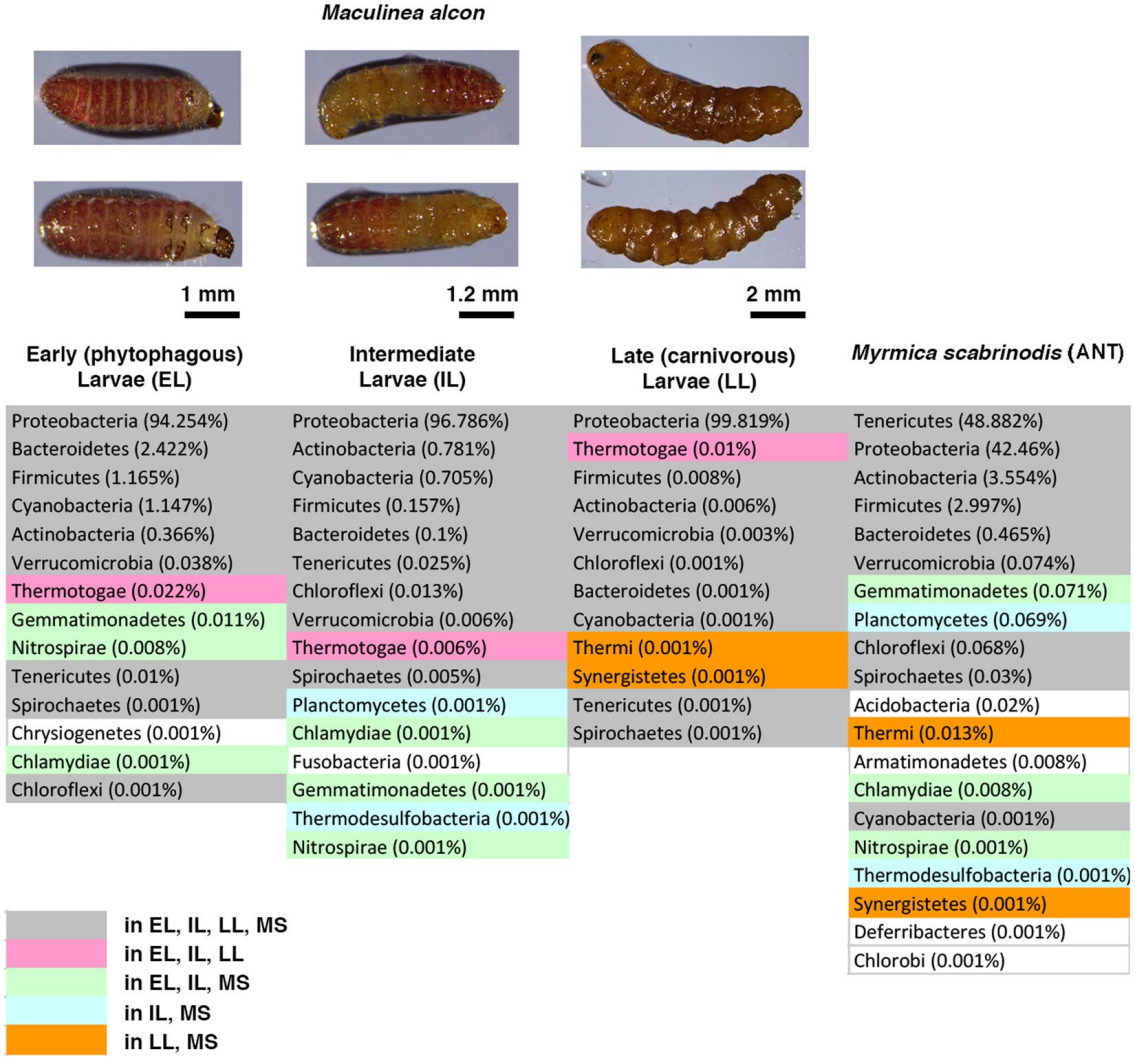
Detected phyla by 16S rRNA gene-based metabarcoding.

Comparative 16S rRNA-based profiling of microbiota of *Ma*. *alcon* instars at different life history stages (phytophagous early larvae [EL], intermediate larvae [IL], carnivorous late larvae [LL]) show small differences between samples in terms of detected phyla. Phyla with a relative abundance equal or higher than 0.001%, are 14, 16 and 12 in EL, IL and LL, respectively (Fig. 1 and Supplementary Dataset S1 and S2). The number of detected phyla is higher, i.e. 20, in the host ant *My*. *scabrinodis*. Several phyla can be found in all samples, while others are detected exclusively in *Ma*. *alcon* (Thermotogae) or in specific larval stages of *Ma*. *alcon* and in *My*. *scabrinodis* (Fig. 1). In particular, Gemmatimonadetes, Nitrospirae and Chlamydiae are detected in both EL and IL (and *M*. *scabrinodis*), whereas Thermi and Synergistetes are only found in LL and *My*. *scabrinodis*. This result indicates dynamic variations of the bacterial community during the larval development of *Ma*. *alcon* from phytophagous EL to carnivorous LL, and possible interactions with the microbiota of the omnivorous ants. During metamorphosis, diet-induced dynamic variability of the “adaptive” microbiota and the presence of common microbiota are reported in other lepidoptera^26–28^.

**Figure 2:**
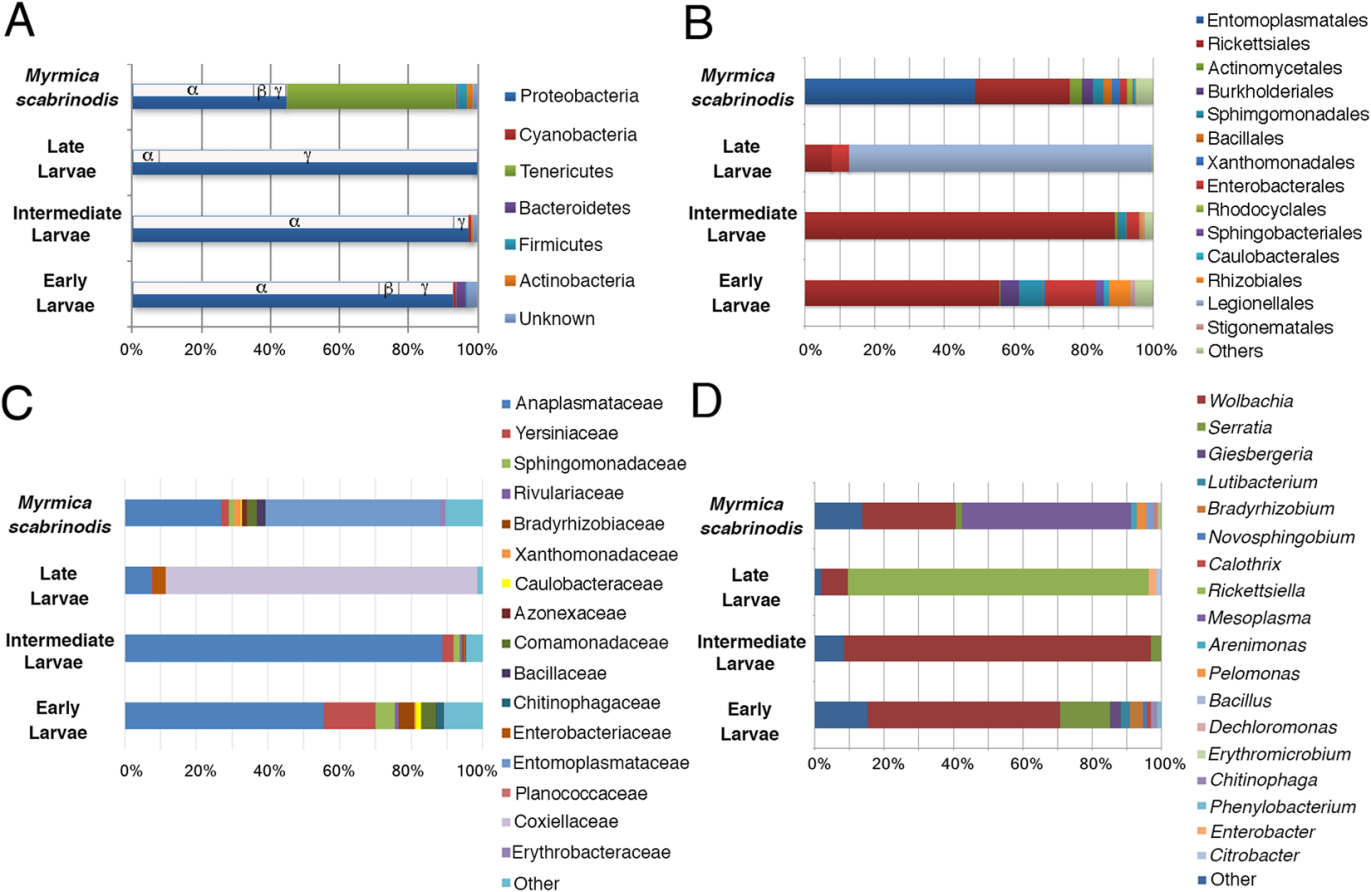
Relative percentages of main phyla (**A**), orders (**B**), families (**C**) and genera (**D**) associated with distinct larval stages and host ants, as deduced by 16S rRNA gene-based metabarcoding. Relative percentages of Proteobacteria sub-phyla are also reported in A.

**Figure 3:**
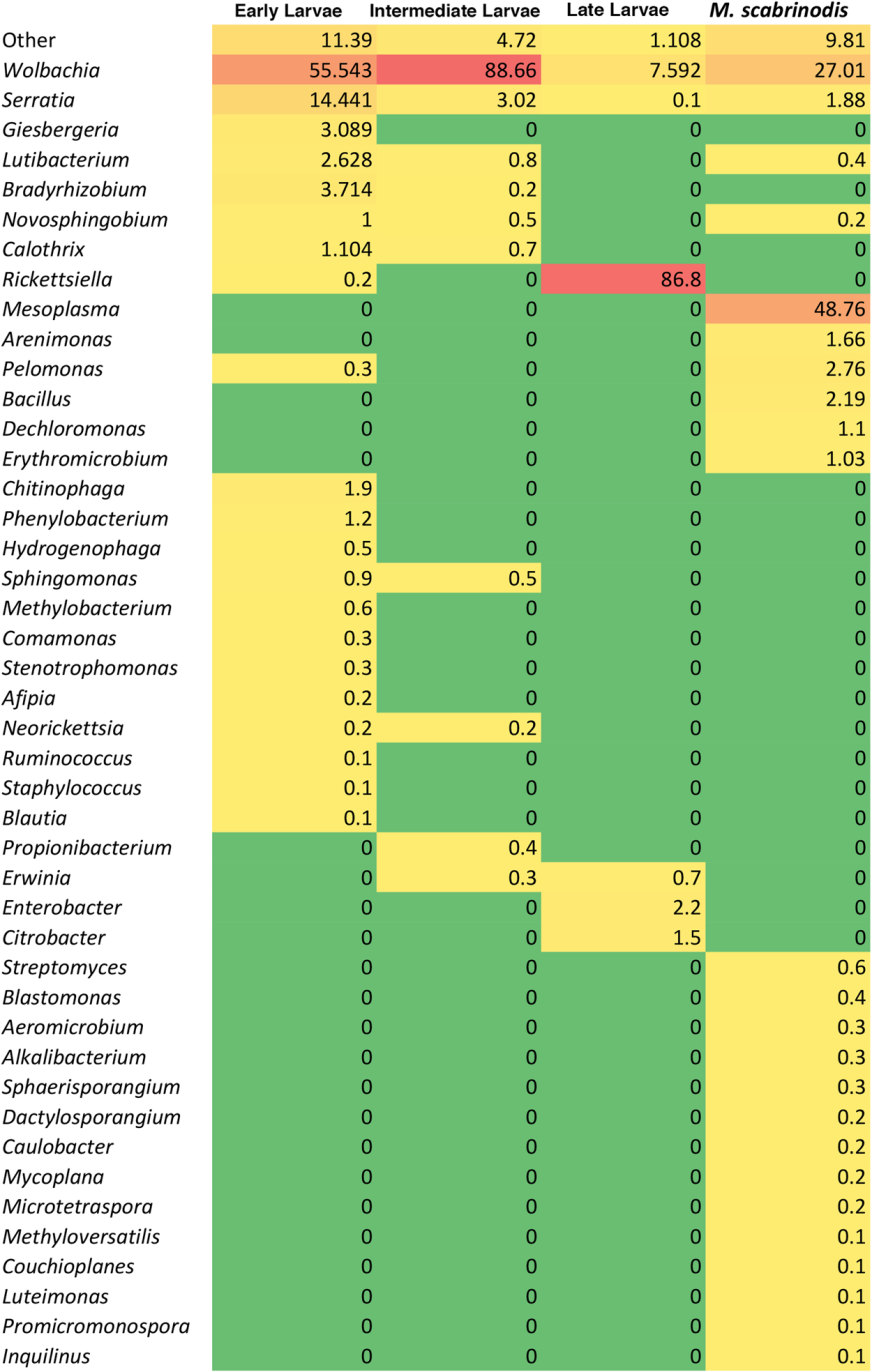
Heatmap showing the distribution of different genera detected by 16S rRNA gene-based metabarcoding among analyzed samples.

Normalized percentages (equal or higher than 1%) of the most representative taxa of bacteria detected are shown in Fig. 2. Proteobacteria dominate the microbiota of *Ma*. *alcon* larvae with normalized percentages of 94.2, 96.8 and 99.8 in EL, IL and LL, respectively (Figs 1 and 2A). Yet in both EL and IL stages Alphaproteobacteria dominate (69% and 91%, respectively), while Gammaproteobacteria dominate the microbiota in the LL stage (92%) (Fig. 2A). Most Alphaproteobacteria in EL and IL stages are Rickettsiales of the genus *Wolbachia* (family Anaplasmataceae) (55.5% and 88.7%, respectively). These maternally transmitted bacterial endosymbionts are well known as reproductive manipulators altering sex ratios in several arthropods^29^ (Figs 2B–D and 3). The presence of *Wolbachia* is well documented in both ants and butterflies^30,31^.

Notably, among Enterobacterales, only bacteria of the genus *Serratia* (Yersiniaceae) are detected in all larval stages of the butterfly and in the host ants (Figs 2B–D and 3). *Erwinia* (Erwiniaceae) is found in both IL and LL, whereas *Enterobacter* and *Citrobacter* (both Enterobacteriaceae) are only detected in LL (Figs 2B–D and 3).

Other less represented groups of bacteria are reported in Fig. 3. From IL and LL microbiota show a substantial reduction in the relative abundance of *Wolbachia* (7.6% in LL), and an increasing prevalence of Gammaproteobacteria of the genus *Rickettsiella* (facultative insect endosymbionts belonging to the family Coxiellaceae of the order Legionallales), which attains normalized percentage of 86.8% (Figs 2B–D and 3).

The prevalence of *Rickettsiella* spp. in LL is noteworthy, because these facultative insect endosymbionts are demonstrated to be responsible for a red to green color shift within a population of pea aphids, that camouflages aphids with leaves of different colors making them less visible to predators and parasites^32,33^. It would be interesting to investigate whether similar a red to yellow color shift, which has been observed during *Ma*. *alcon* larval development is involved in the integration of butterfly larvae in host colonies considering that most ant species have a dichromatic color vision system, which are insensitive to red light^34^.

The metabarcoding analysis of the *My*. *scabrinodis* microbiota shows a slight prevalence of Tenericutes (48.9%) of the *Mesoplasma* genus (Entomoplasmatales, Entomoplasmataceae) with respect to Proteobacteria (42.4%) mostly belonging to Alphaproteobacteria of the genus *Wolbachia* (27%) (Figs 1 and 2). The prevalence of *Mesoplasma* is intriguing because recent evidence suggests that a monophyletic clade of *Mesoplasma* has been found associated with eight Attine, Leaf-Cutter ant species in Brazil and *Atta texana* in the US, but also with Army ants, *Aenictus* spp. and *Eciton* spp.^35,36^.

### Structure similarity of the bacterial communities

**Figure 4:**
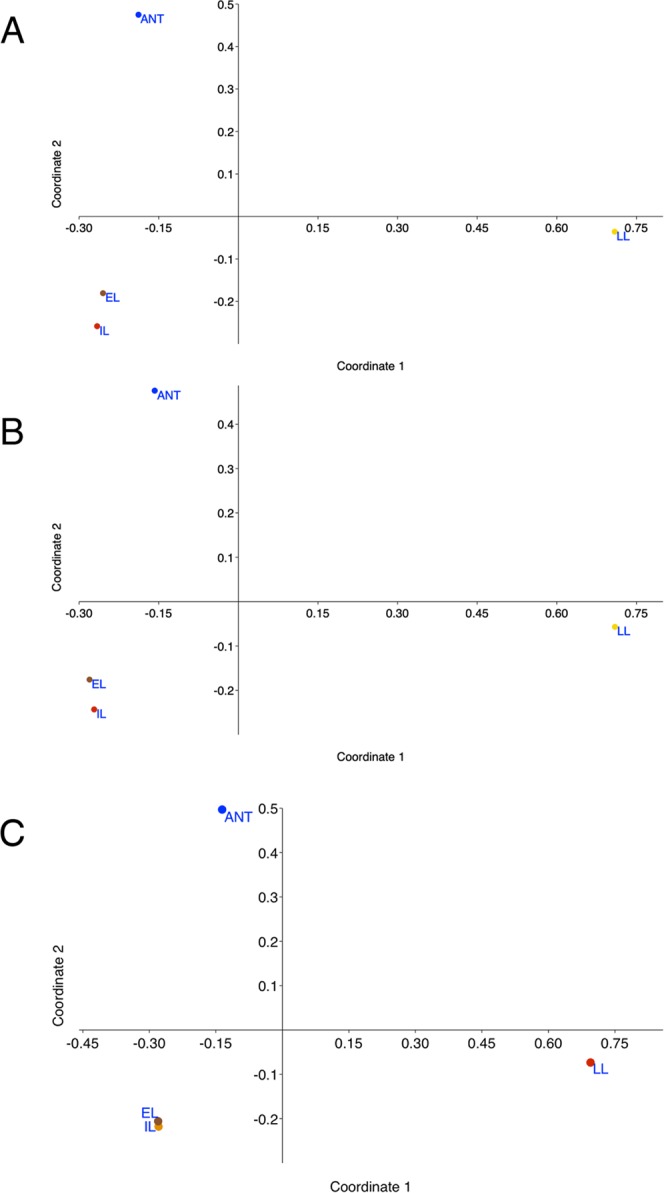
Similarities in the microbiomes of larval stages and host ants. Non-metric Multidimensional Scaling (NM-MDS) analysis of order (**A**), family (**B**) and genus (**C**) data (within-sample abundance). The results of the analysis show global similarity between *M*. *scabrinodis* (ANT) and *M*. *alcon* larvae in early (EL), intermediate (IL) and late (LL) stages. IL and EL stages have a large global similarity. LL is distant as ANT from EL/IL.

Ordination plots of bacterial phylotypes and relative within-sample abundance reveal a complex structure of the bacterial community. NM-MDS (Fig. 4A–C and Supplementary Dataset S3A) indicates a high similarity between EL and IL, which occupy an intermediate position in the plot with respect to *My*. *scabrinodis* and LL. Chart plots are used to obtain a global view of orders, families and genera based on within-sample abundance (Supplementary Fig. S1). The results illustrate that EL exhibits the highest levels of phylogenetic diversity and variance in the relative abundance of the different phylotypes, followed by ANT, IL and LL.

**Figure 5:**
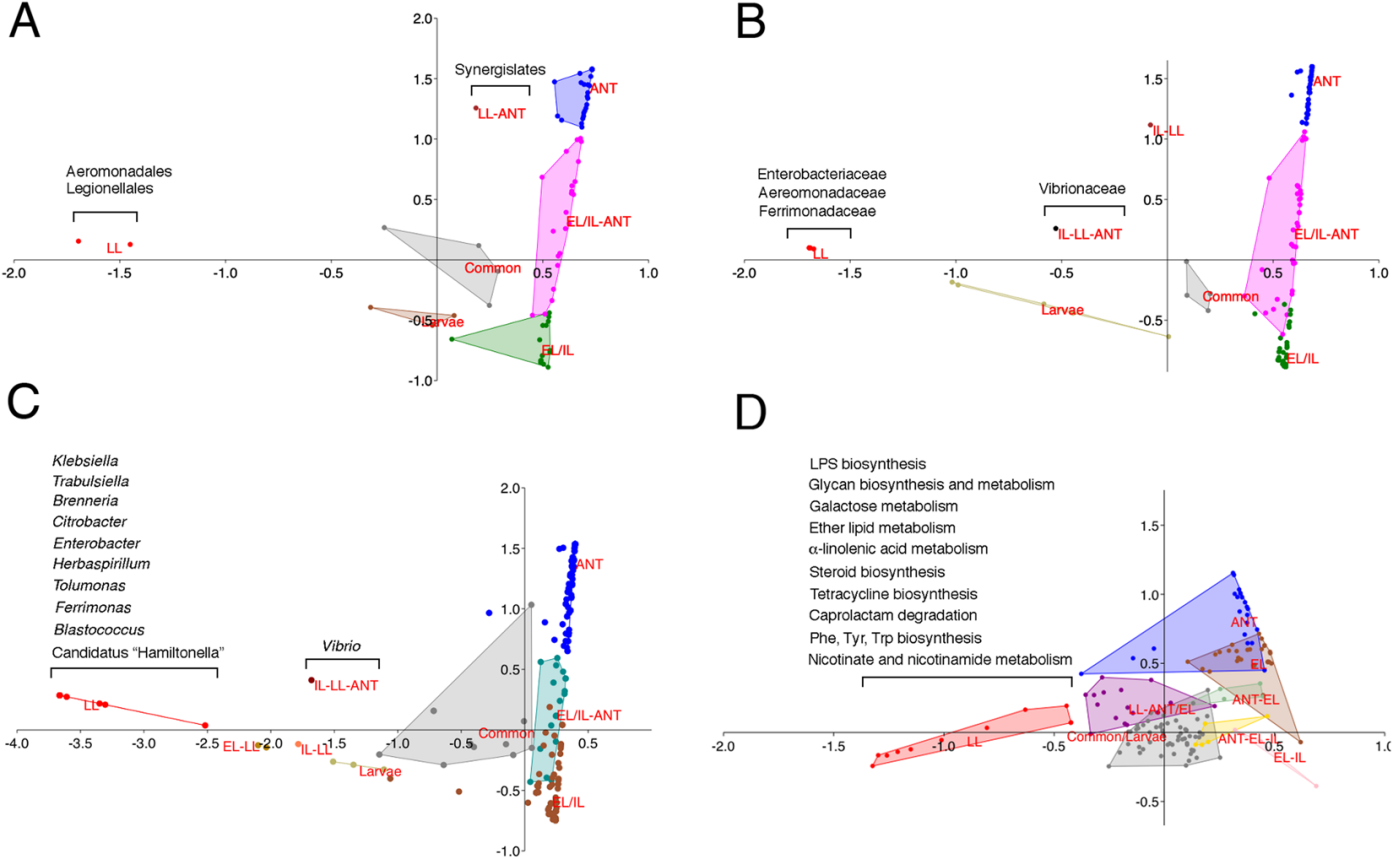
Correspondence analysis of the microbial communities visualized by ordination plot. Data of orders (**A**), families (**B**), genera (**C**) and predicted KEGG pathways by PICRUSt (**D**) were used as input. Orders, families, genera and KEGG predictions that were exclusive or largely predominant in a given sample, were shared between two or three samples, or were common to all samples are shown.

Figure 5 shows ordination plots of a correspondence analysis that highlight groups of taxa exclusive or predominant in a given sample, shared by two or three samples, or common to all samples (Supplementary Dataset S3C). At the level of genera (Fig. 5C), obligate intracellular bacteria are excluded, because they are overrepresented in some samples masking other relevant differences between samples. Only few taxa are found common to all samples (Common), ANT (ANT) or EL, IL and LL (Larvae), while major groups of orders, families and genera are specifically associated with EL and IL (EL/IL), or EL, IL and ANT (EL/IL-ANT). In contrast to our initial hypothesis, we find a very limited overlap between LL and ANT samples. The overlap is restricted to Synergistales (Fig. 5A), which are typically characterized by strong amino acid fermenting capability^37^. Among families, Vibrionaceae is enriched in IL/LL-ANT (Fig. 5B) and genus *Vibrio* is prevalent in IL/LL-ANT (Fig. 5C).

In terms of alpha-diversity, the *Maculinea* microbial community becomes increasingly simpler during larval development (Figs 1–4). This could indicate a secondary loss of endosymbiotic bacteria that in a protected, stable and food rich environment, such as the nest, are not necessary anymore. This might explain why we do not find a close match in the composition between LL and *Myrmica* ant microbiota (Fig. 5), as hypothesized. However, this general pattern does not exclude that bacteria belonging to Thermi or amino acid-fermenting Synergistetes phyla, which are only shared by LL and ants, could be key factors in promoting the digestion of molecules present in the ant regurgitations. Together with further behavioral and morphological adaptations, the ability to share the same diet of *Myrmica* ants would have been essential for *Maculinea* to evolve such a peculiar lifestyle (trophallaxis), which enables the parasite to have this most intimate interaction with its host.

With regard to obligate intracellular genera, which are separately computed, correspondence analysis indicates that some genera are common to all categories sampled and prevalent in EL (*Wolbachia*, *Neorickettsia*, *Ehrlichia*), while other genera are mostly associated with one or two categories (*Rickettsia* in EL and IL, *Rickettsiella* in LL, *Mesoplasma* in ANT) (Supplementary Fig. S2 and Dataset S3B). In insects, endosymbiont shifts or density fluctuations are commonly observed across the different life stages^38^. The endosymbiont plasticity along with the tropism of endosymbiotic bacteria for the different developing tissues may cope with distinct (metabolic and non-metabolic) requirements during the insect development^38,39^.

### Functional microbiomes predictions of bacterial communities

Predicted KEGG pathways by PICRUSt are distributed more evenly among the samples than phylotypes (Supplementary Dataset S4). In many cases, orders, genera and families appear to be enriched or depleted in several samples, while KEGG predictions have a more even distribution among them (Fig. 5D, Supplementary Dataset S3C). This is rather expected, since different bacteria can provide the same or similar functions. PICRUSt predicts KEGG pathways common to all samples, others shared by ANT and EL, and a group of pathways mostly associated with LL (Fig. 5D, Supplementary Dataset S3C). This last group could be split in two subgroups (LL 70 and LL 40–50) based on their relative within-sample abundance (expressed as percentage) in LL with respect to the other samples (Supplementary Dataset S3C). Five pathways, i.e., lipopolysaccharide biosynthesis, lipopolysaccharide biosynthesis protein, glycan biosynthesis and metabolism, steroid biosynthesis, ether lipid metabolism belong to the LL 70 group, and five pathways, i.e., galactose metabolism, alpha-linolenic acid metabolism, tetracycline biosynthesis, caprolactam degradation belong to the LL 40–50 group. The group of KEGG pathways enriched in LL (Fig. 5D) is consistent with the group of the ten genera (Fig. 5C) that, in addition to endosymbiotic *Rickettsiella*, are similarly enriched in LL.

PICRUSt analysis predicts a number of pathways involved with lipid biosynthesis and metabolism that are highly enriched in LL, for instance the ether lipid/fatty acid metabolism and alpha-linolenic acid metabolism (Supplementary Fig. S3A). These functional predictions are noteworthy in light of a possible involvement of the LL-specific microbial community in the synthesis of compounds mimicking ant cuticular hydrocarbons. A number of semiochemicals are identified in mandibular gland and Dufour gland secretions of *My*. *scabrinodis*^40,41^ and the chemical profile of cuticular hydrocarbons (CHCs) of this ant species is described in several studies^42,43^.

The CHC profile of *My*. *scabrinodis* sampled in different European countries is dominated by alkenes (accounting for >50% of hydrocarbon classes), followed by dienes, mono-methyl alkanes, n-alkanes and mono-methyl dienes^43^. Alkane and alkene biosynthesis has been reported in some microorganisms although the biosynthetic pathways are not fully understood^44^. Four major microbial pathways that convert free fatty acids or fatty acid derivatives into alkanes/alkenes have been proposed involving (i.) fatty aldehyde decarbonylation, (ii.) fatty acid decarboxylation, (iii.) head-to-head hydrocarbon biosynthesis, or (iv.) polyketide synthase reactions^44^. Notably, none of these pathways is present in the KEGG database that is used for PICRUSt analysis. Particularly the two-step biosynthetic pathway for alkane/alkene biosynthesis starting from acyl-CoA, and involving acyl-CoA reductase and aldehyde decarbonylase has been better documented than the others^44^. Fatty acid and acyl-CoA reductases are widely distributed in microbial world. In contrast, until a few years ago, decarbonylation of fatty aldehydes is consistently reported only in Cyanobacteria. However, a systematic screening exercise has led recently to the identification of a wide range of microbial species possessing this activity, and in particular, the genus *Klebsiella* is found to have a common ability to produce alkanes from aldehydes via enzyme catalyzed reaction^45^. It would be interesting to test whether this ability is related to the specific association of *Klebsiella* with LL samples. CHCs are the key elements for nestmate recognition processes in social insects. Members of the same colony share a bouquet of CHCs which functions as a template and enables ants to identify intruders^46^^ *and* *references* *therein*^. Therefore, the ability to synthesize CHCs similar to those of the host ant is crucial for the parasite to achieve a full integration in the ant colony, to such an extent that *Ma*. *alcon* larvae are not only accepted, but also treated like ant brood and fed by trophallaxis^43^.

The mechanism underlying the ability of post-adoption *Maculinea* larvae to synthesizes new CHCs peculiar of the host chemical profile is far from understood. The presence of endosymbiotic microbes involved in the biosynthesis and metabolism of lipids suggests that the microbiome could potentially be involved in the *Maculinea* chemical mimicry.

PICRUSt predictions of secondary metabolism pathways also highlight differences between analyzed sample categories. The most marked difference is a group of metabolic pathways that are depleted in IL and more so in LL (Supplementary Fig. S3B). This group included the following pathways: butyrosin and neomycin biosynthesis, penicillin and cephalosporin biosynthesis, carotenoid biosynthesis, stilbenoid, diarylheptanoid and gingerol biosynthesis, beta-lactam resistance, biosynthesis of siderophore group nonribosomal peptides, flavonoid biosynthesis. Another group of secondary metabolism pathways is highly enriched in ants (and highly depleted in LL); this group is composed of the following pathways: caffeine metabolism, sesquiterpenoid biosynthesis, biosynthesis of type II poliketide products, biosynthesis of 12-, 14- and 16-membered macrolides, biosynthesis of vancomycin group antibiotics, betalain biosynthesis, isoflavonoid biosynthesis, indole alkaloid biosynthesis, melanogenesis. Other pathways are specifically depleted in IL and partially enriched in LL, i.e, tetracycline biosynthesis, caprolactam degradation, geraniol degradation. The relative enrichment in pathways for ubiquinone and other terpenoid-quinone biosynthesis and terpenoid backbone biosynthesis in IL is also noteworthy, because they may provide precursors for biosynthesis of farnesene family compounds that are used as semiochemicals by *My*. *scabrinodis*^47,48^.

### Symbiotic *Serratia* spp. as possible sources of deceptive pyrazines

The metabarcoding data indicate the presence of bacteria belonging to the genus *Serratia* in all larval stages of *Ma*. *alcon* as well as in the host ants, *My*. *scabrinodis*. At the species level, *Serratia marcescens* and *S*. *entomophila* are the predominant species, and the relative abundance between them is almost constant in all larval stages of *Ma*. *alcon* (Supplementary Fig. S4). There is experimental evidence that *S*. *marcescens*, a microorganism that is more often recorded in plants and has been associated with growth-promoting activity^49^, is capable of producing volatile pyrazines, including 2,5-dimethylpyrazine and 3-ethyl-2,5-dimethylpyrazine^50^, which are used as pheromones by ants^51^. Production of pyrazines responsible for potato-like odor is also demonstrated in other *Serratia* species including *S*. *rubidaea*, *S*. *odorifera*, and *S*. *ficaria*^52^. Also note that these *Serratia* species cluster together with *S*. *marcescens* and *S*. *entomophila* in the phylogenetic tree^53^. It is possible that the colonization of EL and IL by plant-associated *S*. *marcescens* and *S*. *entomophila*, which is consistent with the feeding behavior of EL and IL eating the host plant, may play a specific role in the production of deceptive pyrazines.

If confirmed, the ability of *Maculinea* larvae to emit the foraging trail markers of its host ant (3-ethyl-2,5-dimethylpyrazine) thanks to the association with *Serratia* spp. would require to re-interpret the observations on the *Maculinea* adoption rituals, which are generally based on caterpillar behavior and tactile signals (CHC).

### Concluding remarks

Our work provides the first description of the microbiota of *Maculinea alcon* and reports how they change during the larval development. The composition and the variation of the microbial community along with larval growth suggest a still uninvestigated role of microbes in driving not only the shift from phytophagous to an omnivorous diet but also in mediating the chemical deception through which the butterfly parasite achieves the integration and exploitation of the host ant society. The microbial communities associated with multicellular organisms have been shown to significantly influence how the latter interact with their environment. Ant and plant evolutionary pathways are tightly related^54^ and provide the basis for the evolution of ant-butterflies symbiosis^55^. Altogether our results suggest the need to consider the structure and function of microbiota as a potential evolutionary force shaping complex and multilevel interactions with the final aim of contributing to their conservation, which is a priority in the case of *Maculinea* butterflies.

## Methods

### Insect collection

Butterflies and ants were collected at Caselette (45°070N, 07°290E), about 20 km far from Turin (NW Italy). In this wet grassland, *Maculinea alcon* exploits *Myrmica scabrinodis* colonies as host and *Gentiana pneumonanthe* as larval food plants. Adult butterflies are on the wing from late July to middle August^56^. Phytophagous *early larvae* [EL] are first instar caterpillars feeding inside the plant buds. To collect EL, gentian buds bearing eggs were gently dissected in the field at the end of the *Ma*. *alcon* flight period. At the beginning of September, five plants with eggs were gathered and maintained in laboratory conditions (26 °C, 60% humidity and 120 mmol m^−2^ s^−1^ light under a 16 L: 8 D regime) until IV instar larvae, called *intermediate larvae* [IL], spontaneously dropped off the plant and were collected. In June, about one month before the adult emergence, *My*. *scabrinodis* nests were excavated and examined for the presence of *Ma*. *alcon* carnivorous *late larvae* [LL]. LLs are overwintering, fully-grown caterpillars, which have been reared by worker ants in the brood chambers for about ten months. Worker ants were collected from the same colonies where *Ma*. *alcon* LLs were found. All samples were immediately soaked in 50% glycerol and stored at −4 °C.

### Sample processing

Whole, surface-sterilized insects were utilized in this study to analyze the dominant microbial taxa mostly associated with the internal body regions. Insects were rinsed in sterile water, soaked in 70% ethanol for 30 s followed by 10% bleach for 30 s, and rinsed again in sterile water. Sterility of insect surfaces was checked by cultural analysis. After surface washing and sterilization, samples were homogenized using sterilized Wheaton^TM^ Dounce tissue grinder in order to release and partially lyse the microorganisms. To minimize inter-individual variations, pools of insects were used for metabarcoding analysis, namely: 20 EL, 40 IL, 5 LL, and 30 ANT.

### DNA procedures

Total DNA extractions from insect homogenized suspensions were performed by the QIAamp DNA Stool isolation Mini Kit (Qiagen) following the manufacturer’s instructions. Eluted DNA was precipitated in ice-cold 100% ethanol and sodium acetate and then resuspended in 10 mM TrisHCl, pH 8. Extracted DNA was sent to Genomix4life S.R.L. (Baronissi, Salerno, Italy) for library preparation and sequencing of the V3 and V4 region of the 16S rRNA gene.

### 16S rRNA gene-based metabarcoding

Next generation sequencing experiments, comprising samples quality control and Bioinformatics analysis, were performed by Genomix4life S.R.L. (Baronissi, Salerno, Italy). Final yield and quality of extracted DNA were determined by using NanoDrop ND-1000 spectrophotometer (Thermo Scientific, Waltham, MA) and Qubit Fluorometer 1.0 (Invitrogen Co., Carlsbad, CA). PCR amplifications were performed with primers: Forward: 5′-CCTACGGGNGGCWGCAG-3′ and Reverse: 5′-GACTACHVGGGTATCTAATCC-3′^57^, which target the hypervariable V3 and V4 region of the 16S rRNA gene. Each PCR reaction was assembled according to 16 S Metagenomic Sequencing Library Preparation (Illumina, San Diego, CA). Libraries were quantified used Qubit fluorometer (Invitrogen Co., Carlsbad, CA) and pooled to an equimolar amount of each index-tagged sample to a final concentration of 4 nM, including the Phix Control Library (Illumina; expected 25%). Pooled samples were subject to cluster generation and sequenced on MiSeq platform (Illumina, San Diego, CA) in a 2 × 250 paired-end format at a final concentration of 10 pmol. The raw sequence files generated (fasta files) underwent quality control analysis with FastQC. The 16S rRNA gene-based metabarcoding analysis performs taxonomic classification of 16S rRNA targeted amplicon reads using the last available version (May 2013) of the GreenGenes taxonomic database. The algorithm is a high-performance implementation of the Ribosomal Database Project (RDP) Classifier described in Wang *et al*.^58^ (http://rdp.cme.msu.edu/).

### Structure similarity and imputed functional predictions of the bacterial communities

For bioinformatic analysis raw data (Supplementary Dataset S1–S4) were refined by calculating WS-A (relative abundance), the resulting data are shown in Supplementary Dataset S3. Starting from WS-A of phylotypes (orders, families and genera) (Supplementary Dataset S1 and S2) and KEGG (PICRUSt^59^ predictions (Supplementary Dataset S4) we clustered the data. To obtain multivariate plots (CA analysis, NM-MDS) and Box-Plots we used PAST 3.18 software^60^. This software package was developed to perform static analysis on paleontological dataset (PAleontological STatistics) but is also useful to analyze biological data. In ordination plots (CA analysis) the tags “EL”, “IL” and “LL” were used to indicate *M*. *alcon* EL, IL and LL larvae respectively, and “ANT” for *M*. *scabrinodis*. For “EL” and “IL” samples, dots in the following plots were very close, so we represented EL and IL in a common cluster tagged with “EL/IL”. To cluster the results and generate plots, BS-A was also determined. In this case, for each genus (or family or KEGG) we summed the values in all samples and divided the value of each genus by the total value. Some order, family and genus that were only present (or largely predominant BS-A ≥80%) in one sample were marked with the sample tag. One group of data showed lower values for “ANT” (BS-A < 10%) and were indicated with tag “Larvae”. Another group of data showed lower value for “LL” (BS-A < 10%) and was labeled “EL/IL-ANT”. Some data were present in all samples (BS-A > 10% for each sample), and marked with the tag “Common”. In the plot of orders (Fig. 5A) one order had a BS-A = 0% for EL and IL and was reported with a “LL-ANT” tag. In the plot of families (Fig. 5B) one family had a BS-A of 0% in EL column and was labeled as “IL-LL-ANT” and another family with BS-A of 0% in EL and ANT columns and were reported as “IL-LL” tag. In the plots of genera (Fig. 5C), two genera had a BS-A = 0 for LL and ANT data (“EL-LL” tag), one had a BS-A = 0 for EL and ANT (“IL-LL” tag) and, finally, one genus had a BS-A = 0 for EL (“IL-LL-ANT” tag). These tags were used to mark clusters of dots in ordination plots. To analyze all data in a global manner we performed a Non-Metric Multidimensional Scaling (NM-MDS) on family and genus data using Bray-Curtis similarity index with samples in rows, and phylotypes (or KEGG pathway) in columns (Supplementary Dataset S3A). In resulting ordination plots samples are represented as dots.

Vice versa, Correspondence Analysis (CA) were performed with samples in columns and phylotype (or KEGG) in rows (Supplementary Dataset S3C). The WS-A of *Wolbachia*, *Rickettsia*, *Rickettsiella* and *Mesoplamsa* were exceptionally dominant compared to other genera (resulting in low-resolution plots) so the data of intracellular obligate bacterial were excluded and the remaining family and genus data were used to generate ordination plots by CA (Supplementary Dataset S3B).

Regarding KEEG predictions, the spread between samples was lower, probably because different strains contribute to predicted KEGG functions, so different thresholds were chosen for clustering (according BS-A: >20, >30, >40, >50 and >70) and the following tagged clusters (with thresholds in parenthesis) were generated: “Larvae”, “Common”, “ANT-EL-LL(20)”, “ANT-EL-IL(20–30)”, “EL-IL(30)”, “EL-LL(30)”, “ANT-EL(30)”, “ANT-LL(30)”, “EL-IL(40–50)”, “ANT(40–50)”, “EL(40–50)”, “LL(40–50)”, “EL(70)”, “LL(70)” and “ANT(70)”. This set of data (Supplementary Dataset S3C) was used to generate CA plots. To simplify plots, groups that showed higher similarity were aggregated: “Larvae” and “Common” were grouped together and indicated as “Common/Larvae”, “EL-LL(30)”, “ANT-EL-LL(20)” and “ANT-LL(30)” were grouped together into “LL-EL/ANT”, “EL-IL(30)” and “EL-IL(40–50)” into “EL-IL”, EL(70) and EL(40–50) into “EL”, “ANT(70)” and “ANT(40–50)” into “ANT”, “EL-IL(30)” and “EL-IL(40–50)” into “EL-IL”, and the remaining groups were reported without threshold value. Data of relevant bacterial orders were extracted and used to construct bar plots and perform CA multivariate analysis.

Information about resources and reagents are provided in the Supplementary Table S1.

## Supplementary information

Supplementary information

Dataset S1

Dataset S2

Dataset S3

Dataset S4

## Data Availability

All data generated or analyzed during this study are included in Supplementary Information files.
