# Supplementary material for: The Microbiome of the Maculinea-Myrmica Host-Parasite Interaction
Source: Sci Rep. 2019 May 29;9:8048. doi: 10.1038/s41598-019-44514-7 (PMC6541603; doi:10.1038/s41598-019-44514-7)
Supplement: Supplementary file 1 — Supplementary information [file 41598_2019_44514_MOESM1_ESM.pdf]

The Microbiome of the *Maculinea-Myrmica* Host-Parasite Interaction

Marco Di Salvo, Matteo Calcagnile, Adelfia Talà, Salvatore Maurizio Tredici, Massimo E. Maffei, Karsten Schönrogge, Francesca Barbero and Pietro Alifano

Supplementary Information

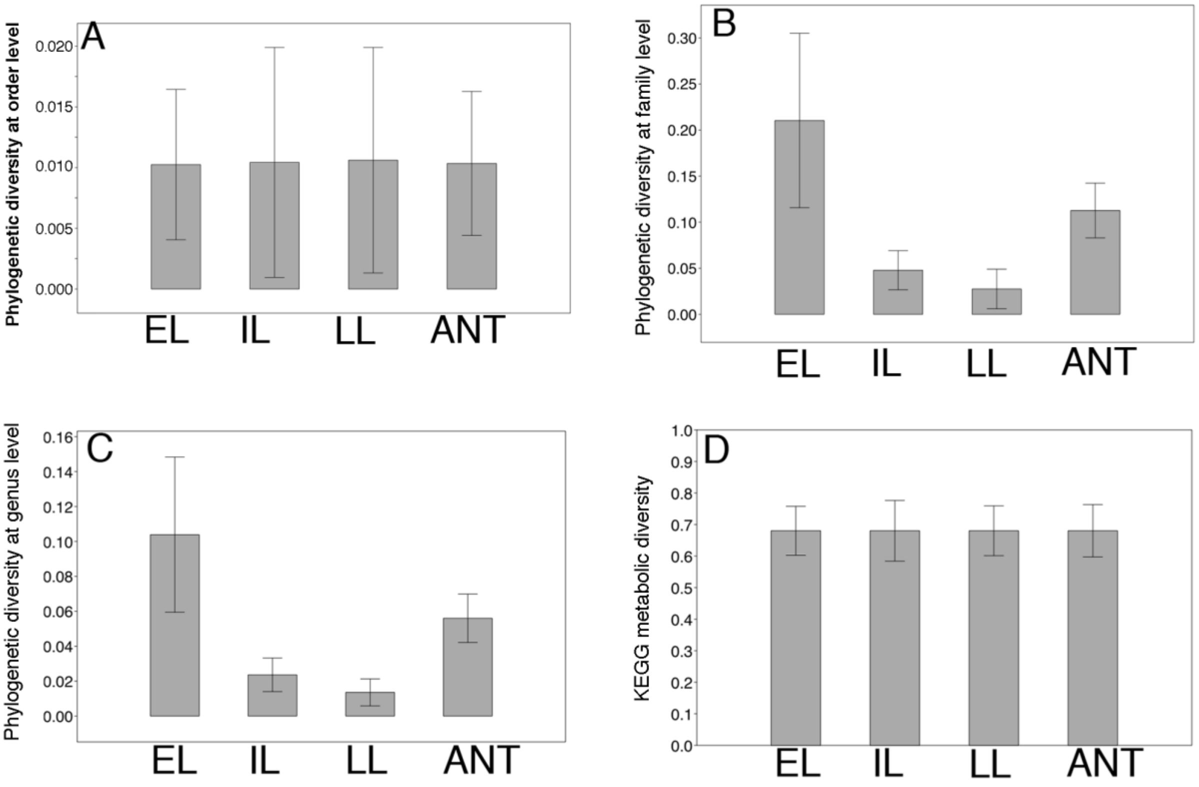

Figure S1. Chart plots analyzing phylogenetic diversity and within-sample variance by using metabarcoding data. Distribution of orders (A), families (B), genera (C) and predicted KEGG pathways by PICRUSt (D) in the different samples. This plot was obtained using PAST software. The mean value of sample is represented as a bar with standard error and a confidence interval of one-sigma (based on standard deviation).

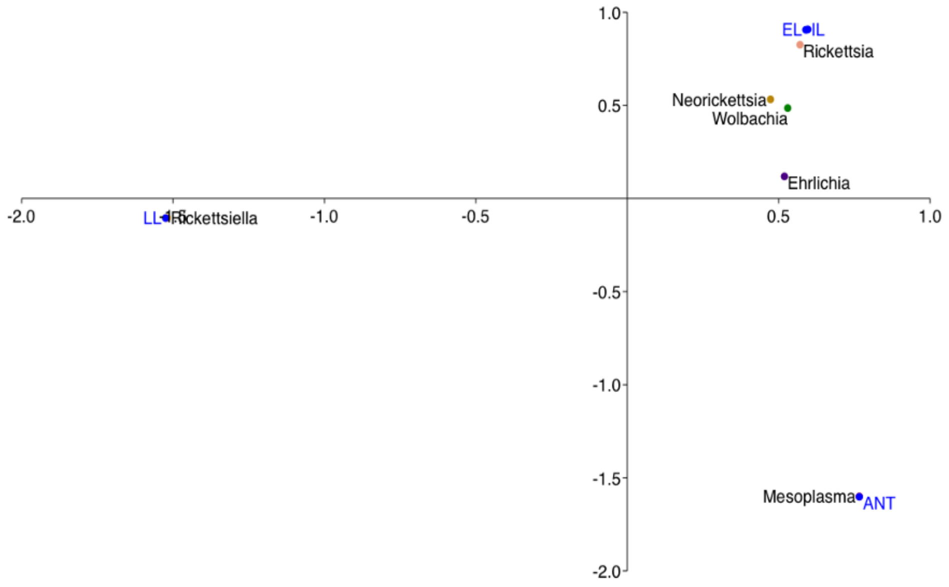

Figure S2. Correspondence analysis (CA), performed by PAST 3. 18 software, of obligate intracellular bacteria (genera, within-sample abundance) based on metabarcoding data. Within-sample abundance of the different genera was used as source.

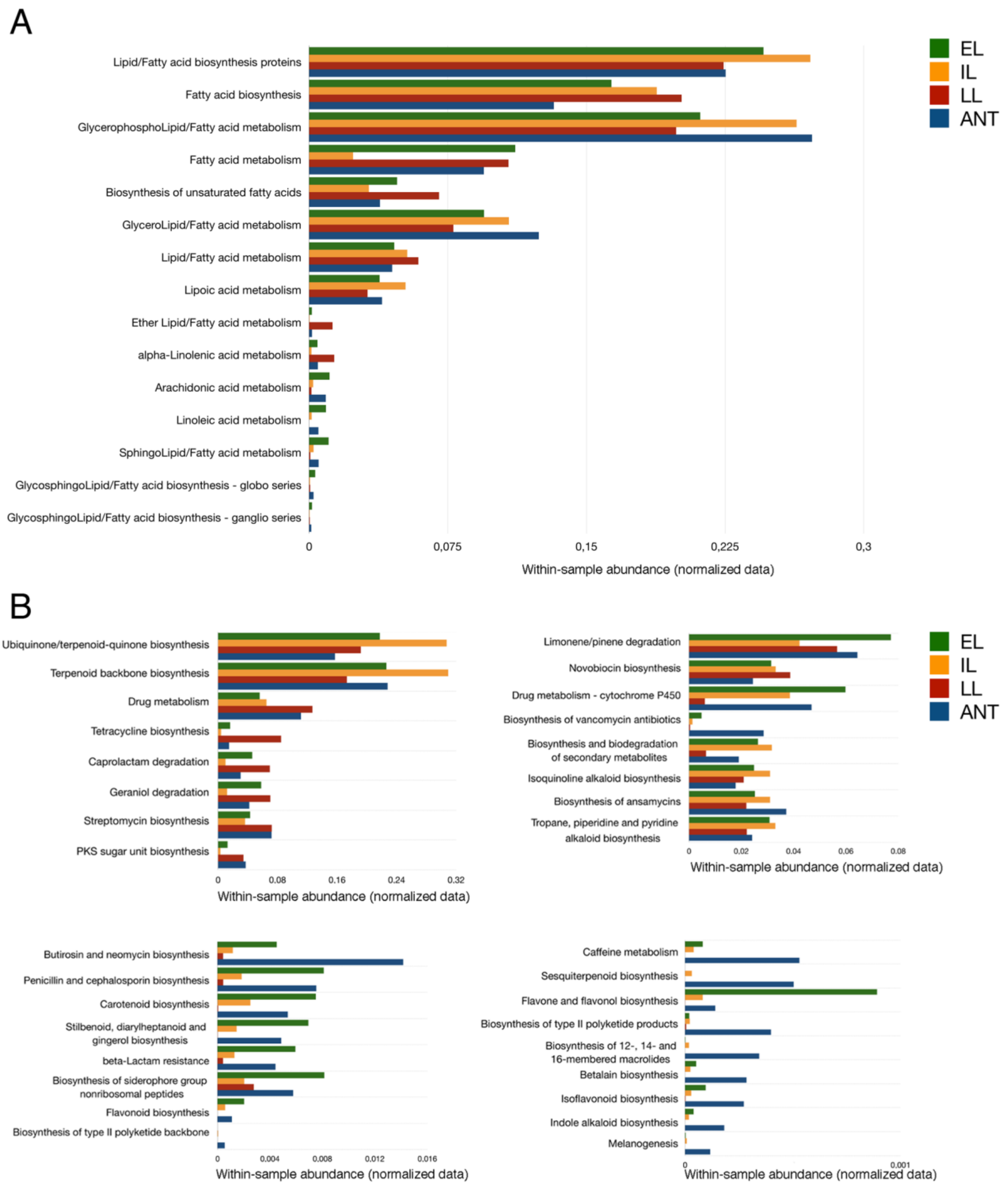

Figure S3. PICRUSt predictions of metabolic pathways enriched or depleted in analyzed samples. Normalized values of within-sample abundance of metabolic pathways dealing with lipid biosynthesis and metabolism (A) and secondary metabolism (B) are shown.

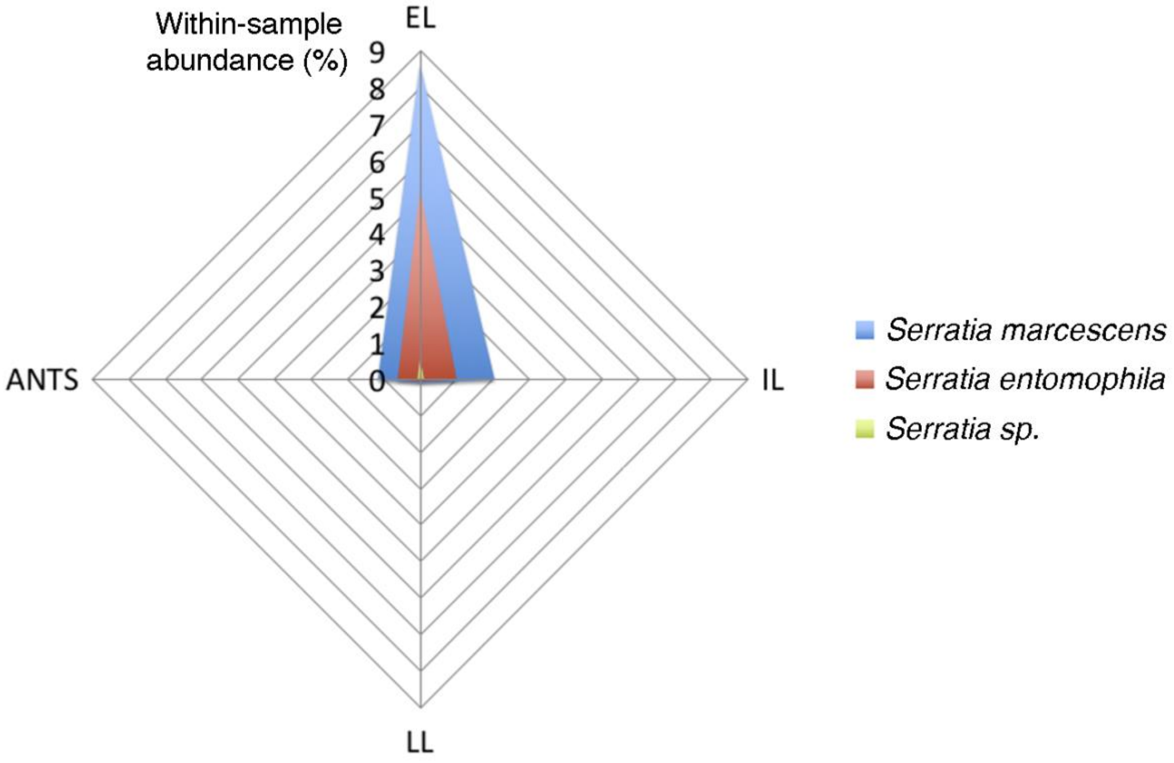

Figure S4. Within-sample abundance of *Serratia marcescens* and *Serratia entomophila* in metabarcoding libraries.

Table S1. Key resources

REAGENT or RESOURCE	SOURCE	IDENTIFIER
**Biological Samples**		
Maculinea alcon larvae	Caselette, Italy (45°070N, 07°290E)	N/A
Myrmica scabrinodis ants	Caselette, Italy (45°070N, 07°290E)	N/A
**Critical Commercial Assays**		
QIAamp DNA Stool isolation Mini Kit	Qiagen	51504
**Oligonucleotides**		
Primer: V3 Forward: 5'-CCTACGGGNGGCWGCAG-3'	Klindworth et al., 2013	N/A
Primer: V4 Reverse: 5'-GACTACHVGGGTATCTAATCC-3'	Klindworth et al., 2013	N/A
**Software and Algorithms**		
Ribosomal Database Project (RDP)	Wang et al., 2007	http://rdp.cme.msu.edu/
PAST 3.18	Hammer et al., 2001	https://folk.uio.no/ohammer/past/
PICRUSt	Langille et al., 2013	http://picrust.github.io/picrust/
**Other**		
16S rRNA gene-based metagenomics	Genomix4life S.R.L. (Baronissi, Salerno, Italy)	https://www.genomix4life.com/en/

References

Klindworth, A. et al. Evaluation of general 16S ribosomal RNA gene PCR primers for classical and next-generation sequencing-based diversity studies. *Nucleic Acids Res.* **41,** doi:10.1093/nar/gks808 (2013).

Wang, Q., Garrity, G. M., Tiedje, J. M. & Cole, J. R. Naive Bayesian classifier for rapid assignment of rRNA sequences into the new bacterial taxonomy. *Appl. Environ. Microbiol.* **73**, 5261-5267, doi:10.1128/aem.00062-07 (2007).

Hammer, Ø., Harper, D. & Ryan, P. PAST: Paleontological Statistics Software Package for Education and Data Analysis. *Palaeontol. Electronica* **4**: 1-9 http://palaeo-electronica.org/2001_1/past/issue1_01.htm. (2001).

Langille, M. G. I. et al. Predictive functional profiling of microbial communities using 16S rRNA marker gene sequences. *Nature Biotech.* **31**, 814-821, doi:10.1038/nbt.2676 (2013).

Dataset S1. Summary of 16S rRNA gene-based metabarcoding data for *M. alcon* early larvae (EL), intermediate larvae (IL), late larvae (LL) and *M. scabrinodis* (ANT) samples.

Dataset S2. Operational taxonomic units **(**OUT) table generated from metabarcoding data.

Dataset S3. Report of bioinformatic analysis of 16S rRNA gene-based metabarcoding data. (Sheet A) This sheet contains three tables for all data of orders, genera and families, respectively. The tags for each sample are on rows, the phylotypes tags are in columns. (Sheet B) Data of intracellular obligate bacteria. (Sheet C) Complete data for orders, genera, families and KEEG predictions. For genus and family data we excluded intracellular bacteria.

Dataset S4. PICRUSt analysis of imputed functional predictions of the bacterial communities.
